# A comprehensive analysis of community pharmacists’ practices, barriers, and strategies in patient education on Saxenda®: a qualitative study

**DOI:** 10.1186/s40780-026-00575-1

**Published:** 2026-04-21

**Authors:** Abdullah Saad Turki, Ehab Mudher Mikhael

**Affiliations:** 1 Al-Rusafa Health Directorate, Ministry of Health, Baghdad, Iraq; 2 Clinical Pharmacy Department, College of Pharmacy, University of Baghdad, Baghdad, Iraq

**Keywords:** Liraglutide, Patient education, Community pharmacists, Obesity

## Abstract

**Background:**

Saxenda® side effects are mostly avoidable, and its administration is complex; therefore, patient education by pharmacists is essential to optimize its efficacy and safety. In Iraq, patient education practices remain inconsistent and barriers to effective patient education are unclear.

**Objectives:**

To evaluate the extent of pharmacists’ practices in patient education regarding Saxenda®, including the adequacy and accuracy of the information conveyed, as well as the barriers encountered in delivering this education.

**Methods:**

A qualitative study was conducted using face-to-face interviews with a purposive sample of community-pharmacists in Baghdad. The interview guide was validated by a panel of experts. Data analyzed by thematic analysis.

**Results:**

Twenty-two pharmacists participated in this study, and three main themes emerged: The first is the extent of current pharmacists’ Practice in patient education, with most pharmacists reporting proactive patient education, though only 13 were consistently proactive. Inquiries about Saxenda® mainly received from young adult women and primarily centered on its effectiveness and side effects. The second theme is pharmacists’ practice in patient education about Saxenda®, where pharmacists frequently educate their patients about the administration techniques and side effects of Saxenda ® verbally, but the information provided was often incomplete or misleading. Most participating pharmacists recognized the benefits of patient education to both patients and pharmacists. The last theme involves the barriers and challenges in patient education about Saxenda® such as patients’ low health literacy and pharmacists’ workload. Participants emphasized the need for targeted training and education to better equip pharmacists with the necessary knowledge and skills for effective patient education.

**Conclusion:**

Community-pharmacists in Baghdad frequently receive inquiries about Saxenda® from young women. Although they recognize the benefits of patient education, their patient education practices remain inconsistent due to barriers such as high workload and limited health literacy. Targeted training, improved resource support, and supportive policies are essential to enhance pharmacists’ skills and address existing barriers in patient education.

**Supplementary Information:**

The online version contains supplementary material available at 10.1186/s40780-026-00575-1.

## Introduction

Obesity is a health problem that occurs when an individual’s energy intake exceeds energy expenditure [1] resulting in increased body fat [2]. Obesity is defined by the WHO as a body mass index (BMI) of ≥ 30 kg/m² [2]. Obesity is a widespread problem, and in 2022, it was reported that over one billion individuals had a BMI exceeding 30 kg/m². Additionally, 43% of the global population is overweight, with Middle Eastern countries exhibiting the highest rates of combined obesity and overweight [3]. Iraq has one of the highest obesity rates in the Middle East region [4]. In this context, a study found that 33.9% of Iraqis were obese, while 31.8% were overweight [5]. The high prevalence of obesity in recent decades may be attributed to the increased availability of energy-dense and unhealthy convenience foods, leading to higher energy intake. Additionally, decreased physical activity and a sedentary lifestyle have contributed to reduced energy expenditure. These factors, combined with genetic predisposition, have resulted in a rise in obesity rates [1].

Obesity is increasingly recognized as a significant health problem worldwide[3]. Several studies found that obesity is associated with increased risk of morbidity and mortality through increasing the risk of cardiovascular diseases, type 2 diabetes, dyslipidemias, osteoarthritis and sleep apnea [6, 7]. To manage obesity and mitigate its associated risks, several medications have been approved, including phentermine, phentermine combined with topiramate, naltrexone with bupropion, orlistat, and glucagon-like peptide-1 (GLP-1) receptor agonists such as liraglutide, semaglutide, and tirzepatide. Among these, GLP-1 receptor agonists are considered the most effective medical treatment currently available for obesity management [8].

Although liraglutide is not the most effective or safest GLP-1 receptor agonist for obesity treatment, its approval for individuals as young as 12 years old and its distinction as the first GLP-1 receptor agonists approved for obesity management [9, 10] have contributed to its widespread availability and use [11].Unfortunately, the administration of Saxenda® is complex, and its numerous side effects require patient education by pharmacists to ensure optimal use [12], which is essential to achieve the full weight loss benefits of Saxenda®[13].

A recent survey of Iraqi pharmacists indicated that while many report providing education on the correct use of GLP-1 receptor agonists for obesity, this practice is inconsistent and not always performed [14]. The study lacks detailed insight into the content, quality, and frequency of such education during routine patient interactions. Additionally, it does not explore the underlying barriers—such as knowledge gaps, systemic constraints, or attitudinal factors—that may impede effective patient education on Saxenda®. Addressing these issues is essential to enhance pharmacist-led patient education and improve obesity treatment outcomes. Therefore, this study aims to evaluate the extent of pharmacists’ practices in patient education regarding Saxenda®, including the adequacy and accuracy of the information conveyed, as well as the barriers encountered in delivering this education.

## Methods

### Study design

A general qualitative study was conducted through face-to-face interviews with pharmacists working at community pharmacies in Baghdad/Iraq, to get in-depth knowledge of the current practices, barrier, and influencers for providing patients with education about Saxenda®.

### Development and validation of the interview guide

The interview guide was prepared by the study authors based on ideas in previous literature [15–18]. The guide was sent to a panel of four academic pharmacists, who have experience in publishing qualitative studies and work in community pharmacy.

The experts were asked to check relevance of each question. Additionally, they were encouraged to provide feedback on any language and wording issues. To assess the content validity of the interview guide, Lawshe’s method was employed [19]. Although all items were considered relevant, comments were received on five items, including recommendations to merge two items and suggestions for linguistic revisions of the other items. After revising the guide accordingly, all experts approved it.

### Study sample

All pharmacists with at least 6 months of working experience in the community pharmacy and have Saxenda® in their pharmacy were considered eligible to participate in this part of the study. A purposive sampling strategy were used to enroll the participants in this study. The recruitment strategy was based on the participants’ academic degree and gender, besides the location of pharmacy to obtain details from different perspectives. All eligible pharmacists were informed about the study objectives and the need to do audio recording. Only those who provided their verbal informed consent were included in the study. Enrolled participants were contacted before the study to allow scheduling a sufficient time for the interview. All participants were interviewed in a quiet area in their pharmacy.

### Data collection and analysis

The interviews were guided by semi-structured open-ended questions (appendix A). Probes were used to elicit further comments when necessary. The interviews were audio-recorded using a mobile recorder. Each interview took approximately 12–20 min. Interviews were conducted until reaching the saturation point, the point at which no new information could be obtained.

The first author transcribed the audio-recorded interviews directly into an Excel sheet in the original Arabic language to ensure no meaning was lost during the data analysis step. Data analysis was done in accordance with Braun and Clarke’s six steps for thematic analysis [20]. The first step involves getting to know the comments by immersing in the data through repeated reading. Next, generating initial codes in English language through the identification and labeling of interesting features within the data. These first two steps were done by the first author; meanwhile, the second author performed a double-check of these English codes against the original Arabic spreadsheet, verifying that the codes accurately represented the participants’ statements to ensure the reliability and ‘trustworthiness’ of the analysis. Any discrepancies in interpretation were resolved through discussion and consensus between study authors. Third, searching for themes by examining these codes to identify broader patterns that capture the main aspects of the data in relation to the research questions. Fourth step involves assessing the generated themes by comparing them against the coded data and the entire dataset to ensure they accurately represent the data and are coherent, and refining them as needed. The fifth step, defining and labeling themes, involves articulating what each theme represents and developing concise, descriptive names that capture their core essence, ensuring clarity and focus. Finally, writing up the results by presenting the themes and supporting evidence in a structured and comprehensive manner. An example of the coding process and the development of themes and subthemes is provided as an additional PDF file [see Additional file 1].

Patient education provided by participating pharmacists regarding Saxenda ® was evaluated in terms of both adequacy and accuracy, in accordance with the information outlined in the Saxenda ® product leaflet. Educational information was considered adequate if it addressed all of the essential educational topics including administration, dosing, side effects, storage of Saxenda®. The educational information was deemed accurate if it aligned with the details specified in the leaflet. Any information provided that conflicts with or contradicts the information in the leaflet was considered incorrect.

## Results

Twenty – two pharmacists participated in the present study. Most pharmacists were males (*n* = 13) and holding BSc degree in pharmaceutical sciences (*n* = 16). The age range of study participants was 26 to 51 years and their working experiences ranged from 2 to 28 years. Further details about study participants are shown in Table 1.

**Table 1:** Demographics of study participants (line 151, page 7)

Number	Age (year)	Sex	Academic degree	Years of experience	Location of pharmacy
1	29	Male	BSc Pharm	6	Suleikh
2	25	Male	BSc Pharm	2.5	Al-Wazireya
3	24	Male	BSc Pharm	3	Meghreb street,
4	29	Male	BSc Pharm-pgy1	4	Al-kasra
5	26	Male	BSc Pharm	4	Al-Fahama
6	25	Female	BSc Pharm	2	Al-karada
7	51	Male	BSc Pharm	28	Saba abkar
8	31	Female	MSc clinical pharmacy	8	Yarmouk
9	30	Male	BSc Pharm	6	Mashatal street
10	33	Male	BSc Pharm-pgy1	10	Meghreb street
11	29	Female	BSc Pharm-pgy1	6	Talbiyah
12	25	Female	BSc Pharm	2	Adhamiyah
13	25	Male	BSc Pharm	4	Palestine street
14	24	Male	BSc Pharm	5	Meghreb street
15	30	Male	BSc Pharm-pgy1	6	Basateen
16	27	Female	BSc Pharm	5	Al-baladiyat
17	27	Male	BSc Pharm	8	Wathiq Square
18	27	Female	BSc Pharm	4	Alameen
19	25	Female	BSc Pharm	2	Suleikh
20	27	Male	BSc Pharm	7	Beirut Square
21	33	Female	MSc pharmacology	11	Adhamiyah
22	26	Female	BSc Pharm	3	Al-baladiyat

Data analysis for this study revealed three main themes (Table 2): Extent of current pharmacists’ practice in patient education, pharmacists’ practice in patient education about Saxenda®, barriers and challenges in patient education about Saxenda®.

**Table 2:** Study themes and subthemes (line 155, page 7)

Study theme	Subtheme
Extent of current pharmacists’ Practice in patient education	Frequency and determinants of pharmacists’ proactive patient education on Saxenda®
	Frequency and triggers for patients’ inquiries about Saxenda ®
Pharmacists’ Practice in Patient Education about Saxenda®	Key Information Typically Provided by Pharmacists When Dispensing Saxenda®
	Methods for delivering information about Saxenda®
	Techniques for assessing patient understanding to educational information about Saxenda®
	Perceived benefits of providing high Quality of education to patients about Saxenda®
Barriers and challenges in Patient Education about Saxenda®	Main challenges facing pharmacist when educating patients about Saxenda®
	Recommendations to improve patient education about Saxenda®

### Frequency and determinants of pharmacists’ proactive patient education on Saxenda®

Most participating pharmacists (*n* = 21) reported being proactive in educating patients about Saxenda®. However, 13 of them indicated that they are proactive at all times when encountering a patient who uses or is prescribed Saxenda®. Three pharmacists stated they are often proactive in patient education. Four pharmacists reported being sometimes proactive in educating patients about Saxenda®. One pharmacist stated that he does so on very limited occasions. On the other hand, another pharmacist reported never providing patient education without a patient request.

For pharmacists who reported not being consistently proactive in patient education, the most common factors influencing their decision to provide education include the use of Saxenda® for the first time (*n* = 7), the patient’s high interest in gaining knowledge (*n* = 1), and patients with a low educational level (*n* = 1). In contrast, factors that hinder pharmacists from being proactive educators include workload in the pharmacy (*n* = 2) and patients being in a hurry (*n* = 1).

*Every time I encounter a patient prescribed Saxenda*®,* I provide them with education. It is impossible for a patient to leave my pharmacy without receiving some form of instruction or information from me.* P17

*I educate about 5 out of 10 patients on Saxenda*®,* often due to the workload in my pharmacy or because the patient is in a hurry.* P 12

### Frequency and triggers for patients’ inquiries about Saxenda®

Although all participating pharmacists reported being asked about Saxenda ® in their pharmacies, only 15 of them were able to specify the frequency of inquiries about Saxenda® in their pharmacies. Six reported receiving inquiries about 2–3 times per month, five indicated receiving inquiries from customers about 1–2 times per week, two stated that requests for advice about Saxenda® occur daily, and the remaining two pharmacists noted that inquiries happen infrequently, approximately once per month or less.

Most participating pharmacists reported that customers seeking information about Saxenda ® is the main trigger for contacting pharmacists in which most patients have multiple questions about Saxenda®. The most common inquiries were related to its effectiveness, specifically weight reduction potential (*n* = 15), side effects (*n* = 12), frequency of use (*n* = 7), cost (*n* = 6), and method of administration (*n* = 5). Additionally, four pharmacists noted that customers inquire about the onset of action and duration of treatment. Two pharmacists reported that customers ask about how Saxenda® compares to other anti-obesity medications. Other reported questions included dosing (*n* = 2), storage (*n* = 1), and injection site (*n* = 1).

According to the participating pharmacists (some of whom reported more than one customer group), most frequent inquiries about Saxenda® came from predominantly female customers (*n* = 21). These included young adult women (*n* = 10), middle-aged women (*n* = 5), elderly obese women (*n* = 4), and women of any age (*n* = 2). Additionally, seven pharmacists reported that diabetic patients commonly asked about Saxenda®. Conversely, five pharmacists noted that obese individuals, regardless of sex, also inquired about the medication. The remaining two pharmacists indicated that adolescents were among those asking about Saxenda®.

Customers especially women aged from 25 to 35 are curious about Saxenda ® side effects, how much weight it can help them lose, and over what time frame they might expect to see results. They also ask how long they should continue using it. In my pharmacy, inquiries about Saxenda ® are repeated to me every two weeks. P18

*Mostly obese patients and those with diabetes ask me about the cost of Saxenda*® and its potential to reduce body weight. Most of these patients are women aged 20s to 30s. In my pharmacy, I receive inquiries about Saxenda® approximately 3 times per month. P16

### Key information typically provided by pharmacists to patients when dispensing Saxenda®

Most participating pharmacists reported providing their customers with multiple information regarding Saxenda®. These included the method of administration (*n* = 19); side effects of Saxenda® (*n* = 19), dosing of Saxenda® (*n* = 14), storage of Saxenda ® (*n* = 9), and the importance of optimum adherence to Saxenda ® (*n* = 2). Meanwhile, none of participants was able to report all these information to the patient.

Regarding patient education about Saxenda® administration technique, nine pharmacists reported providing such information without mentioning any specific details. Only ten pharmacists provided detailed information about what they communicate to their customers in this regard; however, one pharmacist provided incorrect information, and the remaining nine pharmacists did not cover all the essential aspects of Saxenda® administration. The most commonly reported topics included injection site (*n* = 7), changing the needle with each injection (*n* = 5), selecting the dose on the pen (*n* = 5), injection angle (*n* = 2), injection site rotation (*n* = 2), pinching the skin (*n* = 1), holding the needle in the skin for a few seconds (*n* = 1), and the need to prime the pen for the first use (*n* = 1).

Regarding Saxenda® dosing, 19 pharmacists mentioned the prescribed dose to their customers; however, only seven of them educated their customers about dose escalation and only one reported informing their customers about the needed action when missing a dose.

Regarding the side effects of Saxenda®, the most commonly reported ones included nausea and vomiting (*n* = 10), abdominal pain (*n* = 5), gastrointestinal upset (*n* = 2), diarrhea (*n* = 2), lipodystrophy (*n* = 1), and pancreatitis (*n* = 1).

*I educate my patients on how to use the Saxenda*® pen and proper injection site. I also inform patients about the possibility of experiencing nausea and reassure them that this side effect is normal. P11

*First*,* I teach Saxenda*® users how to determine and set the required dose using the pen. I advise them to rotate the injection site from time to time so that inflammation does not occur in the injection area. I also explain how to store the pen and how they can transport it. Additionally,* I emphasize the importance of changing the needle with every use.* P2

Regarding the organization of key information during patient education about Saxenda®, the majority of participating pharmacists (*n* = 14) reported providing tailored education about Saxenda® to their customers. Most of these pharmacists (*n* = 9) customize educational information based on customer questions and needs, while a minority (*n* = 5) tailor the information according to their perception of the customer’s health literacy.

Conversely, more than one-third of participants (*n* = 8) prefer to deliver education in a structured manner, following a specific sequence of information. Among these, most (*n* = 5) prefer to start with information about the administration technique of Saxenda®, while others prefer to begin with details about dosage or storage of Saxenda®. Only three participants provided reasons for their preference to initially educate customers about the method of Saxenda® administration: two aimed to ensure the customer’s understanding of the technique, and one wanted to give the patient time to decide whether they could use the medication. On the other hand, five participants prefer to discuss Saxenda’s side effects at the end of the consultation; however, only one participant explained that this approach helps to avoid frightening the patient with potential adverse effects. Conversely, three participants prefer to delay discussing the required dose until the end of the conversation, but they did not specify reasons for this preference.

*First*,* I teach the patient about the dosage and how to use the medication. Then*,* I inform them that this treatment may cause some side effects and provide them with information about those side effects.* P4

*When I educate patients about Saxenda*®,* I focus on addressing their questions*,* and ultimately*,* we discuss all relevant educational topics and provide the necessary details. I avoid using a fixed or rigid approach*,* as patients’ educational levels differ.* P9

### Methods for delivering information about Saxenda®

All participating pharmacists reported using verbal methods to deliver information about Saxenda® to their patients. However, only three pharmacists relied solely on verbal communication, considering it sufficient for enhancing patient understanding by simplifying and confirming physicians’ instructions. Meanwhile, twelve pharmacists combined verbal education with other educational methods. Of these, seven pharmacists integrated verbal education with written information on the Saxenda® package to ensure better patient understanding and facilitate easier recall of educational information. Three pharmacists preferred to educate patients through verbal methods alongside video demonstrations to improve understanding and make the process more convenient for both the pharmacist and the patient. One pharmacist reported using a combination of verbal education and written brochures to enhance patient recall of educational information. Additionally, another participant provided verbal education along with demonstration videos for patients to watch at home, considering this a standard practice for patient education. Furthermore, seven pharmacists reported combining verbal education with more than one educational method. Six of these pharmacists used a combination of verbal education, written information, and demonstrations, finding this approach easier for pharmacists to simplify educational information and ensure better understanding among Saxenda® users. The last pharmacist combined verbal and written methods and personally administered the first dose to the patient in the pharmacy, aiming to maximize understanding and considering this approach to be more effective and less time-consuming than showing videos.

*Because there are many details*,* I cannot write them all down*,* so I explain everything verbally to patients*,* including the Saxenda*® injection technique and injection sites. Additionally,* I find that patients understand better through this verbal method of education.* P22

*I provide all instructions verbally. However*,* I feel that the patient doesn’t fully understand just by listening*,* so I also write on the package the frequency and dosage. Additionally*,* I always administer the first dose myself so that patients can observe and learn how to inject Saxenda properly.* P16

### Techniques for assessing patient understanding to educational information about Saxenda®

All pharmacists, except one, reported performing certain techniques to assess patients’ understanding of the educational information they provide about Saxenda® in their pharmacies. The most commonly reported method was asking the patient to repeat the educational information(*n* = 13). Meanwhile, four of these pharmacists stated that they also observe patients’ body language while asking about their understanding of the educational information, in order to assess comprehension through verbal and non-verbal cues. Another technique reported to be used by seven pharmacists was direct inquiry, where they simply asked patients if they understood the educational information provided. Meanwhile, five of these pharmacists stated that they also observe patients’ body language while asking about their understanding of the educational information, in order to assess comprehension through verbal and non-verbal cues. On the other hand, two of the above pharmacists reported to further ask their patients if they are in need to repeat the educational information.

The frequency of using these techniques to assess patients’ understanding varied among pharmacists. Eleven pharmacists reported that they always assess their patients’ understanding of the educational information regarding Saxenda®. Three pharmacists indicated that they assess their patients only on limited occasions, skipping the assessment when they perceive that the patient understands the educational information(*n* = 1) or when patients come for medication refills (*n* = 2). Another three pharmacists assess patients’ understanding approximately 80–90% of the time, skipping the assessment if they believe the patient has good health literacy (*n* = 2) or if they are managing a high patient load in the pharmacy (*n* = 1). Additionally, two pharmacists reported conducting the assessment in about 75% of cases; one stated that he only assesses first-time users, while the other mentioned that he sometimes forgets to perform the assessment.

The last two pharmacists reported assessing their patients’ understanding in only about half of the cases. One of these pharmacists skips the assessment if the patient is actively involved in the educational session, and the other skips it if he perceives that the patient understands the educational information.

*After providing education about Saxenda*®,* I ask the patient to repeat the information back to me. I do this with most patients*,* except for those who have used it before.* P18

*I ask the patient if they understand the instructions or if they need a repetition*,* and I pay attention to their facial expressions. If their response is confident*,* I assume they have understood and I do not repeat it. I do this action with all patients* P11

### Perceived benefits of providing high Quality of education to patients about Saxenda®

Participating pharmacists reported numerous benefits of providing high quality education about Saxenda® to patients; some benefits pertained to the patients themselves, while others related to the pharmacists. Nearly all participating pharmacists (*n* = 21) considered patient education about Saxenda® to be beneficial to patients either by increasing the efficacy of Saxenda® (*n* = 16) and/or reducing its side effects (*n* = 9).Meanwhile, participating pharmacists noted that patient education helped to increase the efficacy of Saxenda® by ensuring accurate administration (*n* = 14), proper dosing (*n* = 10), and correct storage of Saxenda® (*n* = 2), as well as improving patient adherence to the medication (*n* = 3). Conversely, nine pharmacists believed that patient education about Saxenda® was beneficial to pharmacists themselves. They identified multiple benefits, including increased trust in pharmacists and their competence (*n* = 6), an improved reputation for the pharmacist and pharmacy (*n* = 6), and greater patient loyalty to the pharmacy (*n* = 3). In addition, most of the participated pharmacists (*n* = 10) reported observing positive outcomes in their patients after educating them on the usage of Saxenda®. These outcomes are either direct, evidenced by witnessing significant weight reduction (*n* = 6), or indirect, through patients returning to the same pharmacy requesting refills (*n* = 2), expressing gratitude to the pharmacist (*n* = 1), or insisting on receiving further education from the same pharmacist who initially provided the education(*n* = 1).

*Patient education generally enhances trust between the patient and the pharmacist*,* improves the pharmacist’s image in the patient’s eyes*,* and encourages the patient to continue returning to the pharmacy where the pharmacist works.* P7

*Patient education ensures that the patient will use Saxenda*® correctly,* continue its use*,* and thereby achieve the maximum benefit. I recall a patient whose weight was 130 kg; after being educated about Saxenda*®,* his weight reduced to 100 kg.* P18

### Main challenges facing pharmacist when educating patients about Saxenda®

Most participating pharmacists (*n* = 19) reported facing at least one challenge when educating patients about Saxenda®. Of these, 15 pharmacists identified the challenges as primarily related to the patients themselves, including low health literacy (*n* = 5), low educational levels (*n* = 4), misconceptions about Saxenda®(*n* = 2), greater trust in physicians’ expertise regarding education (*n* = 2), skepticism about the benefits of Saxenda®(*n* = 1), and fears related to medication side effects (*n* = 1). Conversely, nine pharmacists viewed the educational environment within the pharmacy as the main barrier to patient education about Saxenda®. All of these pharmacists (*n* = 9) cited patient load in the pharmacy as the primary obstacle. Additionally, three of them reported additional barriers to patient load that were closely related to and/or caused by patient load, such as frequent interruptions by other customers (*n* = 2) and the lack of a dedicated consultation area within the pharmacy (*n* = 1). Additionally, one pharmacist considered the complicated educational information for Saxenda® to be the main challenge he faced when educating patients about Saxenda®.

*The most challenging aspect for me is how to help illiterate patients*,* who cannot read or write*,* understand how to use Saxenda*®. P9

*The presence of many patients in the pharmacy hall who speak to me simultaneously puts pressure on me*,* making it difficult to educate patients effectively. Additionally*,* the current pharmacy design in Iraq does not ensure patient privacy.* P1

### Recommendations to improve patient education about Saxenda®

Although 19 pharmacists reported facing challenges when educating their customers about Saxenda®, only 16 of them provided recommendations to overcome these challenges and hence ensure optimum patient education. These included: repeating educational information to patients (*n* = 6), allocating sufficient time for education despite the patient load in the pharmacy (*n* = 5), enhancing education through the use of aids such as videos and leaflets (*n* = 3), providing patients with their phone numbers to ensure ongoing educational support (*n* = 2), creating a dedicated room for patient education (*n* = 1), asking patients to wait until the patient load decreases (*n* = 1), securing a longer period for educating patients with misconceptions about Saxenda®(*n* = 1), reassuring patients that side effects of Saxenda® are generally mild and may not occur in all patients (*n* = 1), and working to build patient trust in pharmacists (*n* = 1).

*I try to allocate sufficient time for patient education*,* even during busy periods in the pharmacy. What is most important to me is that the patient leaves with a clear understanding of how to take the medication correctly.* P4

*As pharmacists*,* we strive to communicate the medication instructions to patients as effectively as possible. For less educated patients*,* I typically show them a YouTube video in the pharmacy to help them watch and understand the information more clearly.* P14

On the other hand, the participating pharmacists offered multiple recommendations to enhance education services provided by pharmacists to patients using Saxenda®. The majority of pharmacists (*n* = 13) recommended educating their peers through educational lectures, courses, conferences, or online resources, including educational videos shared on social media. Additionally, ten pharmacists suggested providing pharmacists with educational aids to support patient education about Saxenda®, such as posters, brochures, and mobile applications. Furthermore, six pharmacists recommended training programs to enhance pharmacists’ patient education roles and skills.

*Educational lectures*,* seminars*,* or online posts about Saxenda*® are necessary to improve pharmacists’ counseling services for patients using Saxenda®. However,* I believe online posts are the most effective*,* as they can reach the largest number of pharmacists through social media groups dedicated to pharmacists.* P13

*We can create a simple brochure demonstrating how to inject the needle and including other important details. This will allow patients to refer to the brochure later*,* especially if the pharmacist is busy*,* helping them remember the key educational points they need to be informed about.* P14

## Discussion

Given that Saxenda® is an injectable medication widely used across diverse age groups, it requires specialized patient education. While prior research has primarily focused on identifying gaps in pharmacists’ clinical knowledge [14], this study is novel in examining the aspects of pharmacists’ professional behavior in patient education regarding Saxenda® within developing countries, with a particular focus on the Iraqi context. It investigates how the pharmacy practice environment, as well as limited regulations for pharmacists’ roles and responsibilities and limited monitoring of the application of these regulations, influences patient education about Saxenda®. Additionally, the study explores how pharmacists educate patients about Saxenda®, the challenges they face, and their recommendations for enhancing the role of pharmacists in patient education.

The results of the current study showed that nearly half of the participating pharmacists reported that they do not consistently engage in proactive patient education when dispensing Saxenda ®. Supporting this finding, several other studies conducted in Iraq found that most of community pharmacists fail to provide patients with adequate education about their treatment [21–23]. However, such studies differ from the current study by focusing on the general perspective and knowledge of pharmacists about their role in patient education, rather than specifically assessing their proactive practices.

By offering education only when prompted or when time permits, Iraqi pharmacists are acting as passive dispensers rather than active clinical interveners, resulting in a low quality of care. Given the complexity of Saxenda® administration, including the need for dose titration and the potential for numerous side effects, inconsistent education heightens the risk of administration errors, suboptimal weight loss outcomes, and unmanaged adverse effects. This situation underscores a systemic need for mandatory patient education standards and the integration of structured checklists into the dispensing workflow to ensure comprehensive patient support and safety.

The participating pharmacists in the current study indicated that their decision to provide proactive education about Saxenda® to patients is influenced by several factors, including patients’ first-time use of the medication, patients’ high interest in gaining knowledge, and patients with a low educational level. On the other hand, current study participants signaled workload in the pharmacy and patients being in a hurry as main factors that discouraged them from offering education about Saxenda®.

Similarly, Tadesse and colleagues identified a busy schedule as the primary barrier preventing pharmacists from providing patient education. They also observed that pharmacists do not prefer educating patients with prior knowledge about their medications [17]. Additionally, being proactive in educating patients with low level of education seems to align with general recommendations to enhance efficacy and safety of medications for such patients [24].

The tendency of pharmacists to provide proactive patient education primarily to first-time or less-educated patients, especially during periods of low workload, may suggests that pharmacists perceive patient education as a discretionary task rather than a fundamental professional responsibility. Consequently, this approach leads to inconsistencies in the delivery of proactive education, and such services may be deprioritized in favor of workflow demands to expedite dispensing during busy pharmacy hours. Therefore, it is highly recommended to raise pharmacists’ awareness of the importance of proactive patient education and to implement clear policies that reinforce the responsibilities and roles of pharmacists.

The present study showed that all participating pharmacists reported receiving frequent inquiries about Saxenda® from their pharmacy customers. This finding was consistent with the picture seen among community pharmacists in Lebanon in which all of them reported being receiving queries about weight management products at their pharmacy [25]. The frequent inquiries to pharmacists about Saxenda® is somewhat expected since it reflects the increased public demand for anti-obesity medications within the region, driven by high obesity prevalence [3–5] and the strong influence of social media marketing [26]. Consequently, it is imperative that pharmacists are equipped with specialized clinical knowledge to address the high volume of inquiries regarding Saxenda®. Such preparation is essential to ensure the rational, safe, and technically accurate use of GLP-1 receptor agonists in the community setting.

Regarding the inquiries about Saxenda®, the majority of pharmacists in the current study reported that such inquiries primarily focused on its effectiveness, potential side effects, frequency of use, and cost. Similarly, Auerbach and colleagues [27] found that effectiveness and side effects are the main factors influencing patients’ decisions to initiate GLP-1 receptor therapy. Questions about the frequency of Saxenda® use are understandable, considering that many individuals fear injections due to its route of administration [28]. Additionally, it is expected that customers seek information about the cost of Saxenda®, as it is an expensive medication. Evidence from the United States indicates that individuals with high out-of-pocket expenses are less likely to initiate GLP-1 receptor agonists [29]. In Iraq, Saxenda® for obesity treatment is not provided through the public sector, where medications are generally free; instead, patients must purchase it directly from private pharmacies [30]. Therefore, it is anticipated that many patients inquire about Saxenda’s cost, as this barrier may significantly influence their decision to start treatment.

The current study revealed that most inquiries to pharmacists about Saxenda® came from female costumers, with the young adult women representing the largest group. This finding aligns with the results of a systematic review, which showed that weight loss attempts are more common among young women [31]. The tendency to seek weight loss medications among women is highly expected, as obesity is more prevalent in females than males [32]. Additionally, women are more likely to perceive themselves as overweight compared to men [33]. Cultural and media pressure for thinness further motivates women to seek weight loss options, which may also contribute to high inquiry rates for Saxenda® among women [26].

On the other hand, some participating pharmacist reported that diabetic patients frequently inquire about Saxenda® in their pharmacies. This finding is not surprising, as liraglutide is a medication with dual action: aiding weight loss and controlling diabetes [27].

Regarding the information typically provided by pharmacists to patients when dispensing Saxenda®, most participating pharmacists reported providing their customers with information regarding the administration, side effects, and dose of Saxenda®; however, the extent of this information varied among pharmacists and was generally poor. This indicates inconsistency and deficiencies in patient education practices, which may be attributed to factors such as varying levels of knowledge, training, or resources among pharmacists. Similar factors have been identified in a systematic review assessing the pharmacist’s role in educating patients about dietary and herbal supplements [34]. Therefore, most participating pharmacists in the current study recommended providing proper training and education to pharmacists to enhance their competence and skills in patient education. Additionally, they emphasized the importance of supplying pharmacists with sufficient resources, including educational aids like brochures and posters. Similar recommendations were reported in previous literature [35], emphasizing their significance and the global need to address these issues in order to enhance the pharmacist’s role in patient education worldwide.

Regarding the organization of key information during patient education about Saxenda®, most of the pharmacists participating in the current study reported preferring a tailored approach over a structured one when educating patients about Saxenda®. Those who tailored educational sessions mainly customized information based on patient questions. However, relying solely on tailoring in this way may be insufficient, as it might not cover all essential information [36] and could result from pharmacist negligence or carelessness. A minority of pharmacists reported tailoring education based on their perception of the patient’s educational level. This approach can be beneficial for patients with genuinely low health literacy who require specialized care, and it aligns with patient-centered care principles described in a previous study in Malaysia [37]. However, the main drawback is that these pharmacists’ actions are based on their perceptions and assumptions rather than an accurate assessment of the patient’s actual needs or literacy level. To improve patient education, pharmacists should incorporate formal assessments of health literacy and ensure comprehensive, standardized information is provided to all patients.

On the other hand, a minority of study participants who reported a preference for delivering education in a structured manner tend to start with details about the administration technique, dosing, or storage of Saxenda®, rather than focusing on side effects. This preferred sequence may be anticipated based on the findings of a recent Iraqi study, which demonstrated that pharmacists have greater confidence in their knowledge of administration techniques and storage compared to the side effects of GLP-1 receptor agonists [14]. Additionally, participating pharmacists indicated that they favor this approach to avoid frightening patients with Saxenda® side effects.

Regarding the methods for delivering information about Saxenda®, the current study showed that all participating pharmacists relied on verbal communication to educate patients about Saxenda®. However, most reported adopting a multimodal educational approach by supplementing verbal education with written handouts, videos, or brochures to enhance patient understanding, improve recall of information, and make the process more convenient for both patients and pharmacists. The benefits of combining verbal explanations with visual aids are well-supported, as this approach addresses different learning preferences, reinforces key messages, and reduces misunderstandings. Ultimately, this enhances proper medication use, increases its effectiveness, and leads to better patient outcomes. Numerous related studies have demonstrated that using a multimodal (verbal and visual) educational approach—particularly for medications that require patients to master specific techniques, such as inhalers [38] and insulin injections [39, 40]—can significantly improve patients’ understanding of proper usage and produce substantial clinical benefits. Therefore, it is highly recommended to encourage all pharmacists to adopt a multimodal approach when providing education about Saxenda® and other GLP-1 receptor agonists.

Patient education by pharmacists is not a guarantee to ensure patient understanding, therefore, assessing patients’ understanding of the educational information is necessary. However, the current study found that only half of the pharmacists regularly assessed their patients’ understanding of educational information regarding Saxenda®. The primary barriers to this assessment included pharmacists’ workload and their perceptions of patients. Similar challenges were identified in a systematic review of the teach-back method in healthcare consultations with patients [41]. This finding reveals a significant “comprehension gap” in patient education process. When pharmacists skip the assessment phase due to a high workload or a subjective belief that a patient “looks” like they understand, they rely on assumption rather than verification. It is possible that many pharmacists perceive assessment of patient understanding by feedback techniques as ‘extra work’ that offer no direct financial gain. Rather, these practices, especially in the high workload environment of Iraqi community pharmacies, are often perceived as detrimental to their business, as they can cause delays in the dispensing process and hence result in the loss of patients who prefer to avoid lengthy waits. In the context of a complex injectable like Saxenda®, this is particularly hazardous. This suggests that for many pharmacists, the goal of patient education is the delivery of information rather than the confirmation of knowledge. Therefore, pharmacists should adopt patients’ assessment methods to optimize education process.

Regarding the patient assessment method, the most commonly reported methods were asking patients to repeat educational information, followed by direct inquiries. Similarly, a previous study in Iraq found that pharmacists employed comparable methods, although they preferred direct inquiry over feedback techniques [42]. The difference between that study and the current one may be due to the fact that, in the current study, pharmacists used these methods to actively assess understanding rather than simply indicating their personal preferences. This reflects their awareness of the complexity involved in patient education regarding Saxenda®, which necessitates the use of more effective techniques to evaluate understanding. Consequently, most pharmacists reported using feedback techniques rather than direct inquiries, as feedback is considered the most effective method for enhancing patients’ knowledge and outcomes [43]. In contrast, direct inquiries are unreliable, since patients may often respond with “yes” to avoid embarrassment [44].

The results of the present study showed that pharmacists considered patient education about Saxenda ® to be beneficial to patients and to pharmacists themselves. Regarding patient benefits, the pharmacists believed that education about Saxenda® improves its efficacy by promoting proper administration, appropriate dosing, and enhanced patients’ adherence. These beliefs are well-supported by existing literature [45, 46] and are further corroborated by the pharmacists’ observations of significant weight reduction in most patients.

Conversely, many of the participating pharmacists believe that patient education can benefit pharmacists financially by directly increasing patient loyalty to the pharmacy, and indirectly by enhancing their professional reputation and strengthening patients’ trust in their expertise and hence patients returning to the same pharmacy requesting refills. This perspective is supported by previous studies, which identified patient education as a crucial service that increases patients’ visits to the pharmacy [47] and, consequently, has the potential to boost pharmacy business profitability [48].

The current study revealed that most participating pharmacists faced several challenges when educating patients about Saxenda®. Some of these challenges are patient-related such as limited education and health literacy, while others are pharmacist-related like high workload and the absence of a dedicated consultation area. Similar obstacles were identified in an Iraqi cross-sectional study assessing the general perceptions and attitudes of community pharmacists towards patient education [21]. Moreover, such challenges have also been reported by Indonesian pharmacists providing education services to diabetic patients [49]. These findings suggest that such challenges are common across different healthcare environments and are not unique to the current context.

Unfortunately, most of the aforementioned challenges—such as limited health literacy and the lack of consultation areas—impede effective communication. Limited understanding may prevent patients from fully grasping the medication’s use and benefits. Additionally, the physical environment of Iraqi pharmacies, particularly the high patients load and the lack of dedicated consultation area, combined with patients’ hurried attitudes and frequent interruptions by other patients create a high-pressure environment that significantly dictate how education is delivered. These high-pressure settings might force pharmacists to accelerate their workflow to maintain dispensing speed. As a result, proactive patient education and assessment techniques like the “teach-back” method may be offered only when the patient prompts the pharmacist or when the workload temporarily permits. The pressure to maintain dispensing speed is further complicated by the nature of the treatment. Due to the inherent complexity of the educational information required for Saxenda® which presents a challenge to pharmacists particularly in high workload settings, pharmacists tend to focus on technical administration while neglecting critical details like dose titration and how to manage missed doses. This behavioral response may be a strategy to manage the volume of information within a limited timeframe, but it results in incomplete patient education. By narrowing the scope of the patient education to just the administration of the injection, essential safety and efficacy information is lost. Finally, patient related factors such as low health literacy, low educational level and misconceptions about Saxenda® further complicate the education as these barriers may increase educational session time and may leads to selective educational practice to save time in a busy environment. As a result, pharmacists might omit essential details or skip assessing patient understanding. To address these barriers, some study participants recommended the systematic use of brochures and written handouts. These tools are essential to ensure that the patient receives complete educational messages even during busy hours or if they are in a hurry.

On the other hand, most participating pharmacists in the present study had suggestions for overcoming barriers to patient education. The most frequently reported suggestion was to repeat educational information to patients by allocating sufficient time for education. This approach is highly recommended for all pharmacists, as repeating educational information has been shown to be an effective strategy for enhancing patients’ understanding and recall of information, particularly in patients with low health literacy [50]. Furthermore, many participants recommended enhancing pharmacists’ clinical roles by strengthening their professional knowledge and technical skills through targeted educational and training interventions.

These interventions should be integrated into the curriculum of the College of Pharmacy in Iraq and reinforced throughout the professional lifecycle through comprehensive continuous medical education (CME) programs. This dual-layered approach has significant implications for advancing pharmacy practice in Iraq.

The findings of the present study had several limitations. The study sample was drawn from a single governorate, which may limit the generalizability of the results to other regions or populations. However, this limitation is minimized because the sample was from Baghdad, the capital of Iraq and the most populous governorate, which allows it to represent a substantial and diverse segment of the Iraqi population, providing insights that are broadly relevant at the national level. Second, participants’ responses, including their perceptions and self-reported behaviors, may be biased by social desirability, potentially leading to over reporting of positive practices or attitudes, hence, potentially affecting the accuracy of findings. Anyhow, this limitation is inherent to the qualitative study design and is commonly observed in qualitative research conducted among Iraqi pharmacists [51–53].

Third, translating quotations from Arabic into English poses the risk of losing or misinterpreting language nuances and cultural contexts, which could affect the precision of results. Meanwhile, such linguistic challenges are common in studies conducted in non-English speaker countries [51, 53]. Fourth, the majority of the participating pharmacists were relatively young and possessed limited professional experience, which may have influenced their perspectives and responses. This demographic characteristic might limit the diversity of viewpoints and affect the comprehensiveness of the findings. Fifth, Patient education was not fully assessed, as patient understandability was difficult to measure, given that the communication skills of pharmacists could not be evaluated through interviews alone without observing actual patient education interactions. Last, it is important to note that this study was qualitative and exploratory in nature; therefore, further quantitative research with larger, more diverse samples is needed to validate and extend these findings.

Nonetheless, this study provides a unique contribution to the existing literature by offering insights into healthcare delivery to obese patients in developing countries, exemplified by the Iraqi situation. The findings highlight that current practices are largely “discretionary,” with pharmacists’ provision of educational information influenced mainly by their subjective assessments of patients’ literacy levels and workload, rather than following standardized clinical protocols. The lack of standardized dispensing guidelines in the Iraqi community pharmacies creates an institutional environment where patient education remains informal and intuitive, which is why inconsistent practices for patient education were observed in the present study. These insights emphasize the urgent need for developing and implementing legal and educational regulations to improve consistency and quality of care. Such interventions lay the foundation for transitioning from informal, intuitive practices to a structured, mandatory framework that ensures safe, equitable, and effective medication use for all patients.

In conclusion, community pharmacists in Baghdad frequently receive inquiries about Saxenda® and its effectiveness, predominantly from young women. While pharmacists recognize the importance of patient education, their practices vary in consistency and influenced by factors such as workload, patient literacy, and individual judgment. Key barriers to effective patient education include high workload, environmental limitations, and low health literacy. Addressing these challenges through targeted educational initiatives, improved resource support, and supportive policies for pharmacists could enhance the quality of patient education, ultimately leading to better patient outcomes and elevated professional standards within the Iraqi healthcare system.

## Supplementary Information

Below is the link to the electronic supplementary material.

Supplementary Material 1

## Data Availability

Data generated and analyzed during the current study are available from the corresponding author upon request.

## Appendix A

1. Based on your experience, which patient groups are most likely to inquire about Saxenda® (e.g., older patients, females, first-time users, or those with comorbid conditions)? Additionally, what is the most common information they seek, and how frequently do these inquiries occur in your pharmacy?
2. When dispensing Saxenda®,how often do you proactively provide education about Saxenda® without a patient request? What factors influence your decision to offer counseling (e.g., patient’s prior knowledge, time constraints)?
3. What key information do you typically provide to patients when dispensing Saxenda®, and in what way do you present such information, please explain?
4. Which methods do you use to deliver information about Saxenda® to your patients (e.g., verbal, written, digital)? Explain why you choose this method?
5. Can you describe the methods or techniques you use to assess your patients’ understanding of the educational information you provide about Saxenda®, and if you do not routinely assess their understanding, could you explain the reasons for this?”
6. What are the common challenges you face when educating patients about saxenda®? And how to overcome these challenges?
7. In your opinion, what are the main benefits of providing education to patients about Saxenda®? Have you observed any specific positive outcomes in patients after counseling, and could you provide an example??
8. What are your recommendations to improve counseling service given by the pharmacists to patients using Saxenda®? Prompts: (e.g., training of pharmacists, changing the current curriculum, give the pharmacists educational aids, etc.)?
9. Do you have any further comments to add?
