# Supplementary material for: A comprehensive analysis of community pharmacists’ practices, barriers, and strategies in patient education on Saxenda®: a qualitative study
Source: J Pharm Health Care Sci. 2026 Apr 21;12:43. doi: 10.1186/s40780-026-00575-1 (PMC13097631; doi:10.1186/s40780-026-00575-1)
Supplement: Supplementary file 1 — Supplementary Material 1 [file 40780_2026_575_MOESM1_ESM.pdf]

Supplementary table 1: An example for the process of generating codes, subthemes and themes.

Theme	Subtheme	Interview Question	Codes Generated	Quotations
Extent of current pharmacists' Practice in patient education	Frequency and determinants of pharmacists' proactive patient education on Saxenda®	When dispensing Saxenda®, how often do you proactively provide education about Saxenda® without a patient request? What factors influence your decision to offer counseling (e.g., patient's prior knowledge, time constraints)?	**Frequency of being proactive in patient education:** Always (n=13); Sometimes [30-50%] (n=4); Often [60-90%] (n=3); Rarely (n=1); Never (n=1). **Influencing Factors:** First-time use (n=7); High workload (n=2); Patient interest in receiving education (n=1); Low educated patients (n=1); Patient in a hurry (n=1).	"Every time I encounter a patient prescribed Saxenda®, I provide them with education. It is impossible for a patient to leave my pharmacy without receiving some form of instruction or information from me." P17 "I educate about 5 out of 10 patients on Saxenda®, often due to the workload in my pharmacy or because the patient is in a hurry." P 12 "I proactively counsel about 90% of patients. The remaining few mention that they use it before." P 6
	Frequency and triggers for patients' inquiries about Saxenda ®	Based on your experience, which patient groups are most likely to inquire about Saxenda® (e.g.,	**Patient groups:** Women (n=21); Young adult women (n=10); DM patients (n=7); Obese individuals	"Customers especially women aged from 25 to 35 are curious about Saxenda ® side effects, how much

		older patients, females, first-time users, or those with comorbid conditions)? Additionally, what is the most common information they seek, and how frequently do these inquiries occur in your pharmacy?	(n=5); Middle-aged women (n=5); elderly obese women (n=4); Adolescents (n=2).<br><br>**Information sought:**<br><br>Weight reduction potential/Kilos (n=15); Side effects (n=12); Frequency (n=7); Cost (n=6); Usage/Method (n=5); Time to lose weight (n=4); Duration of use (n=4); Effectiveness compared to other anti-obesity treatment (n=2); Dose (n=2); Injection site (n=1); Storage (n=1).<br><br>**Inquiry Rate:**<br><br>Daily (n=2); 1-2 times/week (n=5); 2-3 times/month (n=6); infrequently/Once monthly (n=2); Not mentioned (n=7).	weight it can help them lose, and over what time frame they might expect to see results. They also ask how long they should continue using it. In my pharmacy, inquiries about Saxenda ® are repeated to me every two weeks." P18<br><br>"Mostly obese patients and those with diabetes ask me about the cost of Saxenda® and its potential to reduce body weight. Most of these patients are women aged 20s to 30s.In my pharmacy, I receive inquiries about Saxenda® approximately 3 times per month." P16
